# 40 years of AETE: the contribution of scientists and practitioners to the progress of reproductive biotechnologies in Europe

**DOI:** 10.1590/1984-3143-AR2024-0061

**Published:** 2024-08-26

**Authors:** Cesare Galli, Giovanna Lazzari

**Affiliations:** 1 Avantea and Fondazione Avantea Onlus, Cremona, Italy

**Keywords:** assisted reproduction techniques, biotechnologies, AETE, Europe

## Abstract

This conference celebrates the 40th anniversary of AETE. Over the past 40 years, AETE has served as a forum for scientists, practitioners, and students working in assisted animal reproduction in livestock species. AETE conferences have reflected developments in the field, from basic to applied science, as well as regulatory changes in assisted animal reproduction practices. Europe has led the way in these developments for many years, progressing from artificial insemination, embryo transfer, and cryopreservation to semen sexing, in vitro production of embryos, cloning by nuclear transfer, genomic selection, and the rescue of highly endangered species. These significant contributions were made possible by the support of funding agencies, both at the national and European levels, promoting cooperation between scientists and practitioners. Assisted reproduction, and animal breeding more generally, face opposition from various groups, including animal rights activists, vegetarians, proponents of organic farming, environmentalists, certain political parties, and increasing regulatory burdens. These challenges seriously affect funding for scientific research, the work of practitioners, and the breeding industry as a whole. It is crucial to invest time and resources in communication to remind the public, politicians, and regulators of the achievements in this field and the contributions made to the food supply chain and the care of the rural and natural environment.

## Introduction

Europe has always been at the fore front and a leader in the development of assisted reproduction biotechnologies, more specifically termed ART (Assisted Reproduction Technologies). Lazzaro Spallanzani (Italy, 1729-1799) was the first to perform the first successful artificial insemination in a bitch (Lonergan, 2018) and Walter Heape (United Kingdom,1855-1929) was the first to perform embryo transfer (Betteridge, 2003). Artificial insemination and embryo transfer were, and still are, the cornerstones of reproductive biotechnologies in mammalian reproduction. Following our forebears, many pioneers, both in academia and in practice, stepped in to bring reproductive biotechnologies to what we know and practice today, as well as opening new windows for younger generations to look into the future. This paper will not pretend to provide a complete and referenced review of 40 years of reproductive biotechnologies in Europe; rather, it offers a personal perspective. Having worked for 40 years both in academia and in industry, I will discuss on how science and practice have fed each other, primarily through scientific societies like AETE (Association of Embryo Technology in Europe), whose 40^th^ anniversary we are celebrating this year.

Scientific societies like AETE in Europe, or IETS (International Embryo Technology Society) internationally, as well as national societies, have played an important role in advancing the field by bringing together scientists and practitioners around the table to discuss findings in research laboratories, the needs of the industry and practitioners and, most importantly, creating a forum for students to present their work and network with the community during 2 to 3 days meetings, that always included social events to facilitate this. The never-ending struggle of the Board of Governors of these societies and the program chairs has always been, and still is, to find the right balance to attract both scientists and practitioners. Moreover, an important part of such meetings are the exhibitors, not only because they provided the sponsorship to pay part of the expenses, but primarily to showcase newly developed tools, consumables, disposable and reagents required as the procedures developed and the regulatory requirements dictated.

Forty years ago, much of the work presented and discussed was related with cattle, but over the years, reproductive biotechnologies have widened their application to include other livestock species like small ruminants, pigs, buffaloes, horses, etc. but also endangered and exotic species, genomics selection, stem cells and genome editing.

In the paper celebrating the 30^th^ year of AETE (Thibier, 2014) there is a detailed narration of the birth and evolution of AETE. In this paper I will attempt to outline what European scientists and practitioners have contributed to the advancement of reproductive biotechnologies, not only in Europe but globally, and how they have found in the AETE their home.

## The preamble to Assisted Reproduction Technologies in Livestock

Many of the ARTs in use today were developed in Europe and disseminated around the world through exchange visit of scientists or veterinarians or during conferences. One very well know was the meeting organized by Tim Rowson at the animal research station in Cambridge, UK, in 1972, on the collection and surgical transfer of cattle embryos (Betteridge, 2003). Many of the attendees at this meeting were then the founders of IETS in 1974 in Colorado, where George Seidel also started a strong program at Colorado State University for the collection and transfer of cattle embryos. For about a decade, the focus was on the refinement of superovulatory protocols, which are substantially the same as those used today (Lonergan and Sánchez, 2022), the optimization of the flushing and recovery protocol, and the replacement of the surgical embryo transfer with the non-surgical transcervical method (Wright, 1981). The first successful cattle embryo cryopreservation was also achieved in Cambridge by Ian Wilmut (Wilmut and Rowson, 1973) resulting in the birth of a calf named Frosty II.

Although the practitioners in the decade 1984-1994 were working to improve MOET (Multiple Ovulation and Embryo Transfer) or, more generally, in vivo derived embryos, many research laboratories concentrated on the in vitro production of embryos. Only a few years earlier, in1978, Louise Brown was born following in vitro fertilization performed by Robert Edwards (Steptoe and Edwards, 1978), who conducted his experimental work at the Animal Research Station in Cambridge, again working with bovine oocytes. All these successful events in Europe served as a strong starting point for scientist and practitioners to continue their promising work.

## Multiple ovulation and embryo transfer

The basis for the successful production of embryos in vivo is superovulation, and the understanding of the dynamic of follicular development is necessary to exploit the ovarian reserve of oocytes (Monniaux et al., 1983, 2014; Monniaux, 2012). Much of the work was done at INRA and often presented at AETE conferences. The protocol in the early days relied on the use of PMSG (now called eCG) but it had undesired side effects due to its long half -life (Monniaux et al., 1983; Vos et al., 1994). A better understanding of follicular dynamics and follicular wave synchronization has allowed the optimization and development of more user-friendly protocols, but major advances in number of viable embryos produced have not been achieved (Bo and Mapletoft, 2014). The development and use of recombinant b-FSH did not improve the results over the products extracted from pituitary glands (Wilson et al., 1993). Therefore, practitioners still rely today on pituitary extract of porcine origin (Folltropin, Pluset, Stimufol, 2 of these produced in Europe) or sheep origin (Ovagen) with the limitation that being a purified extract, there is inevitable batch to batch variation affecting their efficacy.

## In vitro embryo production

The birth of the first baby by IVF sparked an interest in animal IVF, especially in livestock species, particularly in cattle. Although the first calf obtained by IVF was born in US using in vivo matured oocytes (Brackett et al., 1982), the practical application required the use of immature oocytes harvested from ovaries and matured in vitro to metaphase II. Several European scientists contributed significantly to the in vitro maturation of livestock oocytes (Fulka et al., 1982) demonstrating oocyte developmental competence (Staigmiller and Moor, 1984) and finally, the culture of viable embryos in vitro (Gandolfi and Moor, 1987).

The potential value of in vitro technology quickly caught the interest of investors and the industry. Operations like Ovamass, associated with University College Dublin in Ireland, and Animal Biotechnology Cambridge on the Huntington Road premises in Cambridge, were established with the aim of producing large number of embryos from beef donors to be implanted into dairy cows. Similar operations were established in the Netherlands, France and Italy. Europe quickly became the leader in the production of embryos from slaughtered animals (Galli and Lazzari, 1996), as witnessed by the data published annually by the IETS Data Retrieval Committee.

The use of ovaries from slaughtered animals was very useful for research and for beef animals. However, from a genetic selection perspective, especially for dairy, it had to be done on live animals. In fact, in the years following the steps performed in the human field to obtain the first IVF baby also veterinarians started to practice ovum pick up on cows. The first attempts to use ultrasound guided follicular aspiration for embryo production in vitro were reported by Callesen et al. (1987) and further developed by Pieterse et al. (1988, 1991). Using a human endovaginal probe adapted for the use in cattle, Pieterse reported a recovery rate of 55%, the repeatability of the procedure and the absence of side effects on the donor cows.

Although the procedures for embryo production in those days still required major laboratory refinements that came later on (Galli and Lazzari, 1996), the basics of OPU described by Pieterse et al. are still the same as those used today by many practitioners. Recovery rates have improved to over 70% due to the use of better ultrasound equipment with 6 or 7 MHz convex array probes that provide a better resolution on smaller follicles or the use of gonadotrophin priming that increases the size of smaller follicles. The OPU technique was initially applied on problem cows that did not respond to superovulation (Kruip et al., 1994; Looney et al., 1994), but it was later applied on a wider scale, including on pregnant cows, heifers and prepuberal heifers. (Galli et al., 2001). It is difficult and often not relevant to make comparisons between different data set since there are so many variables involved, most of which are not even manageable. Beef breeds perform better than dairy, dry cows do better than lactating ones and cows perform better than heifers. In vitro produced embryos cultured in presence of serum and/or co-culture had a reduced cryotolerance. For several years, until the culture media were improved, the surrogate sheep oviduct was used to produce freezable embryos (Rizos et al., 2002; Lazzari et al., 2010).

Associated with suboptimal in vitro culture systems the embryo developing in vitro were responsible for the so-called LOS (large offspring Syndrome), especially when embryos were originating from nuclear transfer and other invasive micromanipulations (Farin et al., 2010). The underlying mechanisms were initially described by Young working in Edinburgh (Young et al., 1998; Lazzari et al., 2002b). Due to the deregulation of imprinted genes, LOS resulted in offspring that was well above average birth weight, including placenta hypertrophy and hydroallantoids causing dystocia at parturition and increased stillbirth rate. Although the incidence of the phenomenon has decreased due to the better culture media devoid of fetal calf serum, it has not completely disappeared.

Although Europe led in the development of the technology and its practical application, in several AI organization and amongst practitioners, it did not follow the global trend whereby in vitro produced embryos are rapidly replacing in vivo derived ones today according to IETS data retrieval committee. In Europe, two-thirds of the bovine embryos produced still come from MOET, and in vitro produced embryos are mainly used by bull testing organizations.

Another species where in vitro embryo production is impacting breeding programs and practitioner activities is the horse. The implementation of ICSI (IntraCytoplasmic Sperm Injection), another technique developed in the human field (Palermo et al., 1992) to bypass male infertility, found application in the horse to bypass zona hardening of the oocytes matured in vitro collected from the slaughterhouse or by ovum pick up (Lazzari et al., 2002a; Galli et al., 2007). ICSI is revolutionizing the horse breeding industry because of difficulties in capacitating the stallion spermatozoa, the low quality of many frozen semen samples or the limited availability of semen of dead stallions in addition to the sub-fertile or old mares unusable by conventional in vivo flushing (Lazzari et al., 2020; Claes and Stout, 2022), where once again Europe is leading the way. According to the AETE Data Retrieval Committee, the number of in vitro produced horse embryos is greater than the one produced by in vivo flushing. This is also supported by the competitive advantage that ICSI embryos can be cryopreserved very successfully, both by slow freezing or vitrification, making it possible for a seasonal breeder like the horse to produce embryos also outside the breeding season for transfer during the breeding season or for marketing. The pregnancies and the foals obtained from ICSI embryos are normal and do not exhibit phenotypical abnormalities like the LOS observed in ruminants. This technique is also rapidly developing both in South and North America, as it has for bovines.

## Embryo/semen sexing and Genomic selection of livestock

Having the offspring of the desired sex has always been the desire of all breeders. When PCR came on the market in 1988, sexing of cattle embryos became a reality for many cattle breeding organization, especially in Europe for dairy breeds (Bredbacka et al., 1995; Thibier and Nibart, 1995), and also for individual practitioners when portable kits and simplified protocols were developed for field use. The procedure required to take a biopsy from the embryos (5 to 10 cells) had to be done carefully w/o damaging the embryo too much, and the embryos and fresh transfer was the preferred protocol. Embryo sexing is also used in horses where there is no sexed semen available at the commercial level (Lazzari et al., 2020; Coster et al., 2023). While embryo sexing allows to know the sex of the embryo, the use of sexed semen predetermines the sex of the embryo allowing the production of the desired sex only. The refinements and the commercialization of the semen sexing technology was later done in US but the initial groundbreaking experiments on separating X and Y sperm were performed by Jane Morrell (current Board Member of AETE) at the National Institute for Medical Research in London (Morrell et al., 1988). The technique of embryo biopsy, superseded by sexed semen for sex selection, has remained relevant with the introduction of genomic selection (Hayes et al., 2009). Several cattle breeding organization in Europe were quick to implement genomic selection of embryos before transfer or freezing (Ponsart et al., 2014) to accelerate selection primarily on the male line, to select the bull of the next generation and avoid the birth of unwanted bull calves. Similar work has been undertaken also on equine embryos (Coster et al., 2024).

## Cloning by nuclear transfer

Another dream of the animal breeders was to achieve the quality and uniformity typical of plant breeders where cloning is widely used. Cloning mammals is more complicated and the first experiments were actually performed in amphibians (Gurdon, 1962). Despite attempts by many laboratories to clone mice and a controversial publication by an Austrian investigators (Illmensee and Hoppe, 1981) claiming success, the mouse turned out to be more difficult to clone than livestock.

Steen Willadsen a Danish veterinarian working in Cambridge (an AETE pioneer awardee), was a key player in cloning sheep and cattle both by blastomere separation and by nuclear transfer (Willadsen, 1986). Embryo cloning, as developed by Willadsen, had clear limitations on the number of nuclei available in each morula (20 to 30 cells) used, and the process of serial cloning had limitation after the first round. Despite this, embryo cloning was taken up by newly established cloning companies interested in cattle breeding in North America. However, it became clear that, together with the technical difficulties, the phenotype of the embryo was unpredictable, and the interest waned.

It was again thanks to European scientists, with the cloning of Dolly the sheep (Wilmut et al., 1997) that cloning, or better defined as Somatic Cell Nuclear Transfer, regained attention. The possibility to clone an adult animal of known phenotype clearly makes the difference and re-ignited the interest of the industry as well as scientists. After Dolly several other mammals were cloned from somatic cells in Europe including the bovine (Galli et al., 1999), the horse (Galli et al., 2003), the rat (Zhou et al., 2003), the mouflon through interspecies nuclear transfer (Loi et al., 2001) to mention a few.

Studies were also undertaken, especially by Yvan Heyman (Heyman et al., 2007) to demonstrate that the products originating from cloned animals did not differ from non-cloned controls. Despite all these efforts and the pioneering role of many European scientists, cloned animals and their products are not allowed to enter the food chain in Europe. Cloning by somatic cell nuclear transfer is still not efficient, especially in ruminants but works better in pigs and horses.

The reprogramming of the genome of a differentiated cells provided an unprecedented opportunity for scientists interested in understanding the epigenetic events underlying cell differentiation (Yang et al., 2007; Matoba and Zhang, 2018). The unravelling of the mechanisms involved in genome differentiation and reprogramming will be important to increase the efficiency of SCNT. However, these advancements will probably not come from European scientists or industry since the funding of the EU that supported most of the past European successes described above is no more available and directed to other “politically correct” priorities.

## Stem cells and genetic engineering

In 1981 Martin Evans, working at Cambridge, UK, published a seminal paper to describe the derivation of embryonic stem (ES) cells from the mouse embryo (Evans and Kaufman, 1981). This work earned him the Nobel prize in 2007 shared with Mario Capecchi and Oliver Smithies for the development of “gene targeting”, concept largely used today for genome editing. The use of embryonic stem cells became fundamental to generate the knock out mouse models to understand the function of any given gene in the genome (Robertson et al., 1986).

Given the potential of embryonic stem cells several laboratories attempted to derive ES cells from livestock species (Notarianni et al., 1991) as it would provide an unlimited source of cells for cloning. However, it turned out to be a daunting task (Galli et al., 1994). Interest in stem cells was also driven by the possibility of genetic engineering as it was done in the mouse, but the molecular pathways were only partially understood (Lazzari et al., 2006). The undifferentiated state could only be kept for a limited time in culture and it was not until the conditions for human ES cells were worked out (Thomson et al., 1998) that the derivation of stable bovine ES cells was reported (Bogliotti et al., 2018). Currently the interests in livestock ES cells is mainly academic since cloning can be done with somatic cells and it appears that there is no advantage to using less differentiated cells for nuclear transfer compared to fully differentiated ones (Sung et al., 2006).

The interest in generating livestock carrying genetic modification was present also in Europe after the pioneering work in USA of Brinster (Hammer et al., 1985) by microinjecting the pronucleus of the zygote as it was done in the mouse. Several animals carrying transgenes of pharmaceutical interest were generated (Clark et al., 1989; Niemann et al., 1996) but the efficiency of the system was low making the projects very expensive and, in the long term, unsustainable.

The breakthrough to generate genome edited animals came with the discovery of programmable nucleases in the last ten to fifteen years. First, the Zinc fingers nucleases (Urnov et al., 2010), then the TALENs (Joung and Sander, 2013) and the Crispr/Cas9 (Jinek et al., 2012) opened a new era in the genetic modification of animals and plants. Unfortunately, most of the work behind these developments and applications took place in North America as in Europe the “phobia” against GMOs has cut the funding to scientists, driven away companies and investors. Interestingly, the basic discovery behind the CRISPR/Cas9 technology was done by F.J. Mojica a Spanish scientist working in Alicante (Mojica et al., 1993; Lander, 2016).

On the 25^th^ anniversary of the birth of Dolly (Galli and Lazzari, 2021) we are witnessing a revision of the European policy on these techniques, defined as New Genomic Techniques (NGT) (https://ec.europa.eu/food/plant/gmo/modern_biotech/new-genomic-techniques_en). Someone would say better late than never. This revision is primarily considered for plants but still not for animals despite the vast number of livestock genome edited already generated (Bishop and Van Eenennaam, 2020) for agricultural purposes in many parts of the world except Europe.

Currently in Europe we are using genome editing techniques in the field of xenotransplantation to create pigs whose organs, tissues or cells could be transplanted to humans (Fischer and Schnieke, 2022; Galli, 2023) or to generate animal models of human genetic diseases (Aigner et al., 2010; Porta-Sanchez et al., 2023). All this work is at the R&D phase, as the regulatory pathway for approval through the regulatory agencies has yet to be tested.

## Assisted Reproduction Technologies for conservation biology

An area where ARTs are put at work at its best and Europe is leading the way is for the Biorescue project (https://www.biorescue.org/), a race against time to save the Northern White Rhino, an iconic species where only two female are living on earth and are based in Kenya. This project besides being unique in its scope it will also serve as a template for other endangered species. Several European institutions are involved in this project covering the clinical area on live animals for oocyte recovery and embryo transfer (Leibniz Institute for Zoo and Wildlife Research, Berlin), the politics and logistic (Safari Park Dvůr Králové, Czech Republic), embryo production (Avantea, Cremona), stem cell biology (Max Delbrück Center for Molecular Medicine) and ethic (Department of Comparative Biomedicine and Food Science, Padova). To date the project has been very successful with the initial trials with Southern White Rhino females to develop and validate the technique that produced embryos and two lines of ES cells (Hildebrandt et al., 2018). In 2019 we initiated the OPU and embryo production on the two NWR female left (Najin and Fatu, mother, and daughter). Soon we realized that only the daughter was producing embryos therefore we stopped collecting the mother. To date we have produced and cryopreserved 30 NWR embryos and many more from SWR (Hildebrandt et al., 2023). In the meantime, we have been working to develop embryo transfer. The challenges are many including the preparation of a vasectomized teaser bull to detect exactly when the surrogate recipients are in estrus. To date one pregnancy has been established with a SWR embryo (unpublished). To widen the genetic base to be able to have a self-sustaining population we are also using stem cell technologies to generate oocytes and spermatozoa in vitro through iPSc (induced pluripotent stem cells) (Hayashi et al., 2022; Zywitza et al., 2022). The application of ART in conservation biology is viewed with suspicion by the stakeholders who, for a long time, opposed their introduction. Therefore, it is imperative that an ethical assessment is in place before, during, and after the procedures are performed, both on the animals and in the laboratory (Mori et al., 2021; Biasetti et al., 2022).

## Final considerations

ARTs and related techniques have made huge progress in the past 40 years, both in livestock and wildlife species. This progress has been fostered by several circumstances. First, by the public funding made available at the national and, above all, the European level. This has facilitated the propensity of laboratories to exchange scientists, collaborations between research groups, and presentations of original work at conferences. In the last decade or more, such funding for livestock research is no longer available, hastening competition rather than collaboration, as the driving forces to attract funding are now the number of publications or the number of patents at the expense of innovative, reproducible, and sharable work. Second, by the number of public institutions and practitioners, with companies or cooperatives that created a critical mass of knowledge and work with direct practical implication that required solutions. Third, the scientific societies like AETE with annual meetings fostered the exchange of ideas, discussions with regulators and interactions in presence between members that, with the digital era and the recent pandemic, had suffered a lot.

Looking ahead the prospects are not very optimistic. Alongside the reduction of funding, there is also a growing opposition to animal breeding. This opposition arises not only for ethical reasons but also due to concerns about environmental impact and other trendy topics in today’s political discussions. Unfortunately, these discussions often overlook the role of assisted reproduction in a broader context, including its significance in human fields, which are strongly interconnected. As for the future, I believe it will largely be in the hands of the younger generations. While they are being trained with modern techniques and tools, it's important for them not to forget the lessons of the past. By learning from history, they can better plan for the future using the new techniques and instruments available today.
